# Supplementary material for: Cost-Effectiveness of Treatment Optimisation with Biomarkers for Immunotherapy in Solid Tumours: A Systematic Review
Source: Cancers (Basel). 2024 Feb 29;16(5):995. doi: 10.3390/cancers16050995 (PMC10930512; doi:10.3390/cancers16050995)
Supplement: Supplementary file 1 [file cancers-16-00995-s001.zip › cancers-2801651-supplementary.pdf]

cancers MDPI

Supplementary Materials: Cost-Effectiveness of Treatment Optimisation with Biomarkers for Immunotherapy in Solid Tumours: A Systematic Review

Sara Mucherino, Valentina Lorenzoni, Isotta Triulzi, Marzia Del Re, Valentina Orlando, Annalisa Capuano, Romano Danesi, Giuseppe Turchetti and Enrica Menditto

Supplement Box S1. Search Strategy

Query	Keywords (in Mesh OR Title and Abstract)	PubMed	Embase
#1	Antibodies, monoclonal	234,701	534,564
#2	Immunotherapy	315,085	230,610
#3	(nivolumab/ipilimumab[Title/Abstract]) OR ipilimumab[Title/Abstract]) OR durvalumab[Title/Abstract]) OR atezolizumab[Title/Abstract]) OR nivolumab[Title/Abstract]) OR pembrolizumab[Title/Abstract]) OR "Ipilimumab"[Mesh]) OR "durvalumab" [Supplementary Concept]) OR "avelumab" [Supplementary Concept]) OR "atezolizumab" [Supplementary Concept]) OR "Nivolumab"[Mesh]) OR "pembrolizumab" [Supplementary Concept]	9,625	32,770
#4	Immune checkpoint inhibitor	27,009	22,451
#5	Neoplasms	3,340,811	3,956,642
#6	Cancer	3,785,562	3,707,300
#7	Carcinoma	911,084	1,209,542
#8	Tumor OR Toumor	1,283,887	1,580,661
#9	Target therapy OR Chemotherapy	406,424	772,722
#10	Biomarkers OR PD-1 OR Programmed Death 1 OR PD-L1 OR Programmed Death Ligand 1 OR IL-6 OR Interleukin-6 OR CTLA-4	404,251	565,451
#11	Cost benefit analysis	82,517	60,024
#12	Cost effectiveness	109,292	162,052
#13	Cost utility	4,773	10,928
#14	Economic evaluation	85,211	251,560
#15	Quality of life	327,312	495,744
#16	1 OR 2 OR 3 OR 4	110,915	297,320
#17	5 OR 6 OR 7 OR 8	3,073,490	3,573,285
#18	11 OR 12 OR 13 OR 14 OR 15	386,004	652,103
#19	16 AND 17	71,773	140,761
#20	19 AND 9 AND 10	3,763	10,894
#21	20 AND 18	164	566

Supplement Box S2. Search Syntaxes

PubMed

("antibodies monoclonal"[Title/Abstract] OR "Immunotherapy"[Title/Abstract] OR ("nivolumab ipilimumab"[Title/Abstract] OR "Ipilimumab"[Title/Abstract]) OR "durvalumab"[Title/Abstract] OR "atezolizumab"[Title/Abstract] OR "Nivolumab"[Title/Abstract] OR "pembrolizumab"[Title/Abstract] OR "Ipilimumab"[MeSH Terms] OR "durvalumab"[Supplementary Concept] OR "avelumab"[Supplementary Concept] OR "atezolizumab"[Supplementary Concept] OR "Nivolumab"[MeSH Terms] OR "pembrolizumab"[Supplementary Concept] OR "immune checkpoint inhibitor"[Title/Abstract]) AND ("Neoplasms"[Title/Abstract] OR "Cancer"[Title/Abstract] OR "Carcinoma"[Title/Abstract] OR "Tumor"[Title/Abstract] OR "Toumor"[Title/Abstract]) AND ("target therapy"[Title/Abstract] OR "Chemotherapy"[Title/Abstract]) AND ("Biomarkers"[Title/Abstract] OR "PD-1"[Title/Abstract] OR "programmed death 1"[Title/Abstract] OR "PD-L1"[Title/Abstract] OR "programmed death ligand 1"[Title/Abstract] OR "IL-6"[Title/Abstract] OR "Interleukin-6"[Title/Abstract] OR "CTLA-4"[Title/Abstract]) AND ("cost benefit analysis"[Title/Abstract] OR "cost effectiveness"[Title/Abstract] OR "cost utility"[Title/Abstract] OR "economic evaluation"[Title/Abstract] OR "quality of life"[Title/Abstract]) Sort by: Publication Date

EMBASE

(cost AND benefit AND analysis:ti,ab,kw OR (cost AND effectiveness:ti,ab,kw) OR (cost AND utility:ti,ab,kw) OR (economic AND evaluation:ti,ab,kw) OR (quality AND of AND life:ti,ab,kw)) AND (target AND therapy:ti,ab,kw OR chemotherapy:ti,ab,kw) AND (biomarkers:ti,ab,kw OR 'pd 1':ti,ab,kw OR (programmed AND death AND 1:ti,ab,kw) OR 'pd l1':ti,ab,kw OR (programmed AND death AND ligand AND 1:ti,ab,kw) OR 'il 6':ti,ab,kw OR 'interleukin 6':ti,ab,kw OR 'ctla 4':ti,ab,kw) AND ((antibodies AND monoclonal:ti,ab,kw OR immunotherapy:ti,ab,kw OR (immune AND checkpoint AND inhibitor:ti,ab,kw)) AND [embase]/lim OR ((nivolumab:ti,ab,kw OR ipilimumab:ti,ab,kw OR durvalumab:ti,ab,kw OR atezolizumab:ti,ab,kw OR pembrolizumab:ti,ab,kw OR avelumab:ti,ab,kw) AND [embase]/lim)) AND (neoplasms:ti,ab,kw OR cancer:ti,ab,kw OR carcinoma:ti,ab,kw OR tumor:ti,ab,kw OR toumor:ti,ab,kw) AND [embase]/lim

Supplementary Table S1. Key features of economic methods for the overall included studies

Study Reference, year	Study design	Type of Economic evaluation	Perspective used in the analysis	Type of costs included	Reference year of costs	Sensitivity analysis
Barbier MC et al., 2021 [9]	Markov model	Cost-effectiveness analysis	Switzerland third-party payer	Direct health costs	CHF without reference to the year	One-way and probabilistic
Chang WC et al. 2021 [67]	De novo partitioned-survival model	Cost-utility analysis	Taiwan third-party payer	Direct health costs	2020 New Taiwan dollars (NT$)	One-way
Cheng S et al 2021 [33]	Partitioned survival model	Cost-effectiveness analysis	US and China third-party payer	Direct health costs	2021 US dollars	One-way and probabilistic
Liu X et al 2021 [62]	Partitioned survival model	Cost-effectiveness analysis	Chinese third-party payer	Direct health costs	2021 US dollars	One-way and probabilistic
Liu G et al 2021[34]	Decision-analytic model	Cost-effectiveness analysis	Chinese third-party payer	Direct health costs	2019 US dollars	One-way and probabilistic
Hu H et al 2021 [12]	Markov model	Cost-effectiveness analysis	US third-party payer	Direct health costs	US dollars without reference to the year	One-way
Shi Y et al 2021 [68]	Partitioned survival model	Cost-effectiveness analysis	US third-party payer	Direct health costs	2021 US dollars	One-way and probabilistic

Cai Y et al 2021 [35]	Survival analysis and partitioned survival model	Cost-effectiveness analysis	China third-party payer	Direct health costs	2020 US dollars using the exchange rate: 1 US dollar = 6.4721 Chinese yuan renminbi.	Deterministic and probabilistic
Qin S et al 2021 [47]	Markov model	Cost-effectiveness analysis	US third-party payer	Direct health costs	2021 US dollars	One-way and probabilistic
Peng Y et al 2021 [48]	Markov model	Cost-effectiveness analysis	China third-party payer	Direct health costs	2021 US dollars	One-way and probabilistic
Qiao L et al 2021 [13]	Markov model	Cost-effectiveness analysis	China third-party payer	Direct health costs	2020 US dollars	Deterministic and probabilistic
Yang SC et al 2021 [36]	Markov model	Cost-effectiveness analysis	US third-party payer	Direct health costs	2020 US dollars	One-way deterministic
Wurcel V et al 2021 [56]	Partitioned survival model	Cost-effectiveness analysis	Argentina social security perspective	Direct health costs	2020 Argentinian pesos	One-way deterministic
Insinga RP et al 2021 [11]	Markov model	Cost-effectiveness analysis	US third-party payer	Direct health costs	2019 US dollars	One-way and probabilistic
Peng Y et al 2021 [37]	Markov model	Cost-effectiveness analysis	US third-party payer	Direct health costs	2020 US dollars	Deterministic
Liu Q et al 2021 [14]	Markov model	Cost-Effectiveness Analysis based on the 3-year follow-up	US third-party payer	Direct health costs	2021 US dollars	Univariate and probabilistic
Panje CM et al 2020 [15]	Markov model	Cost-Effectiveness Analysis; lifetime horizon	Switzerland third-party payer	Direct health costs	2019 CHF	One-way deterministic
Wan N et al 2020 [38]	Markov model	Cost-effectiveness Analysis; lifetime horizon	US and China third-party payer	Direct health costs	2018 US dollars	Univariate and probabilistic
Weng X et al 2020 [63]	Markov model	Cost-Utility Analysis; lifetime horizon	US third-party payer	Direct health costs	2019 US dollars	One-way and probabilistic
Weng X et al 2020 [16]	Markov model	Cost-Effectiveness Analysis; 10 years horizon	US payer perspective	Direct health costs	US dollars without reference to the year	One-way and probabilistic

Wu B et al 2020 [64]	Markov model	Cost-Effectiveness Analysis; 10 years horizon	US third-party payer	Direct health costs	2018 US dollars	One-way and probabilistic
Zhou K et al 2020 [57]	Markov model	Cost-utility analysis	US third-party payer	Direct health costs	2019 US dollars	One-way deterministic
Criss SD et al 2020 [39]	Markov model	Cost-utility analysis over 5 years	US third-party payer	Direct health costs	2019 US dollars	Deterministic and probabilistic
Lauren B et al 2020 [69]	Markov model	Cost-utility analysis lifetime	US third-party payer	Direct health costs	2018 US dollars	One-way and probabilistic
Liu Q et al 2020 [40]	Partitioned survival model	Cost–utility analysis	China third-party payer	Direct health costs	2018 US dollars	One-way
Parmar A et al 2020 [54]	Markov model	Cost-effectiveness analysis	Canada third-party payer	Direct health costs	2018 Canadian dollars	One-way and probabilistic
Rothwell B et al 2020 [17]	Partitioned-survival model	Cost-effectiveness analysis	England third-party payer	Direct health costs	2015 English pounds	One-way and probabilistic
Li J et al 2020 [18]	Partitioned survival model	Cost-effectiveness analysis	US third-party payer	Direct health costs	2019 US dollars	One-way and probabilistic
Aziz MIA et al 2020 [41]	Decision-analytic model	Cost-effectiveness analysis	Singapore third-party payer	Direct health costs	Singapore SGD dollars without reference to the year	One-way and probabilistic
Hale O et al 2020 [49]	Partitioned survival model	Cost-Effectiveness Analysis; a time horizon of 15 yr (lifetime)	US third-party payer	Direct health costs	2018 US dollars	Deterministic
Wu B et al 2020 [19]	Markov model	Cost-Effectiveness Analysis; 20 years horizon	US and China third-party payer	Direct health costs	2018 US dollars	One-way, two-way and probabilistic
Patterson K et al 2019 [50]	Markov model	Cost-Effectiveness Analysis; lifetime	Sweden third-party payer	Direct health costs	2018 Euro	One-way and probabilistic
Reinhorn D et al 2019 [53]	Markov model	Cost-Effectiveness Analysis; 10 years horizon	US third-party payer	Direct health costs	2017 US dollars	One-way and probabilistic
Longjiang S et al 2019 [20]	Markov model	Cost-effectiveness Analysis;	US third-party payer	Direct health costs	2016 US dollars	Univariate and probabilistic

		lifetime (30 years)				
Wan X et al 2019 [42]	Partitioned-survival model	Cost-effectiveness and cost-utility analysis over 10 years	US third-party payer	Direct health costs	2018 US dollars	Univariate deterministic and multivariate probabilistic
Zhou K et al 2019 [43]	Markov model	Cost-effectiveness analysis	China third-party payer	Direct health costs	2019 US dollars	Deterministic
Bhadhuri A et al 2019 [21]	Partitioned-survival model	Cost-effectiveness and cost-utility analysis over 20 years	Switzerland third-party payer	Direct health costs	2018 CHF	One-way deterministic and probabilistic
Chouaid C et al 2019 [22]	Partitioned-survival model	Cost-effectiveness and cost-utility analysis; lifetime	France third-party payer	Direct health costs	2017 Euro	One-way deterministic
Criss SD et al 2019 [51]	Microsimulation model	Cost-effectiveness analysis	US third-party payer	Direct health costs	2017 US dollars	One-way deterministic
Huang M et al 2019 [23]	Partitioned survival model	Cost-effectiveness analysis	US third-party payer	Direct health costs	2019 US dollars	One-way and two-way deterministic
Insinga RP et al 2019 [31]	Markov model	Cost-effectiveness analysis	US third-party payer	Direct health costs	2018 US dollars	One-way probabilistic
Liu M et al 2019 [58]	Markov model	Cost-Effectiveness Analysis, duration of 2 years	China and US third-party payer	Direct health costs	2018 US dollars	Probabilistic
Loong HH et al 2019 [30]	Markov model	Cost-Effectiveness Analysis; 1 mo over a 10-yr time horizon	Hong Kong	Direct health costs	Hong Kong (HK) dollars	Univariate and probabilistic
Liao W et al 2019 [44]	Markov model	Cost-Effectiveness Analysis	China third-party payer	Direct health costs	2017 US dollars	Univariate
Meng Y et al 2018 [65]	Markov model	Cost-utility analysis lifetime	England third-payer	Direct health costs	2017 british pounds	Probabilistic
Sarfaty M et al 2018 [52]	Markov model	Cost-utility analysis	US third-party payer	Direct health costs	2016 US dollars	Univariate and probabilistic

Sarfaty M et al 2018 [55]	Markov model	Cost-Effectiveness Analysis; 1 mo over a 5-yr time	US, UK, Canada and Australia third-party payer	Direct health costs	2017 US dollars	Univariate and probabilistic
Tarhini A et al 2018 [66]	Markov model	Cost-utility analysis over lifetime	US third-party payer	Direct health costs	2016 US dollars	Deterministic and probabilistic
Zargar M et al 2018 [59]	Cohort based, state transition model	Cost-utility analysis over 5 years	Canada third-party payer	Direct health costs	2016 Canadian dollars	Univariate and probabilistic
Aguiar P et al 2018 [45]	Markov model	Cost-Effectiveness over 5 years and budget impact	Argentina, Brazil and Peru third-party payer	Direct health costs	US dollars without reference to the year	None
Hirschmann A et al 2018 [60]	Markov model	Cost-effectiveness over 60 months	Switzerland third-party payer	Direct health costs	2018 CHF	Univariate and probabilistic
Hu x et al 2018 [32]	Markov model	Cost-Effectiveness and cost-utility analysis	UK third-party payer	Direct health costs	2017 British pounds	Univariate and probabilistic
Insinga RP et al 2018 [25]	Cohort based simulation model	Cost-utility analysis; lifetime	US third-party payer	Direct health costs	2017 US dollars	Univariate and probabilistic
Georgieva M et al 2018 [26]	Bayesian Markov model	Cost-utility analysis; lifetime	US and UK third-party payer	Direct health costs	2018 US dollars	Probabilistic
Ward MC et al 2017 [61]	Markov model	Cost-utility analysis over 36 months	US third-party payer	Direct health costs	2017 US dollars	Univariate and probabilistic
Aguiar PN Jr et al 2017 [27]	RCT-based model	Cost-utility analysis	South America third-party payer	Direct health costs	US dollars without reference to the year	None
Huang M et al 2017 [29]	Partitioned survival model	Cost-effectiveness and cost-utility analysis; lifetime	US third-party payer	Direct health costs	2016 US dollars	Univariate and probabilistic
Matter-Walstra K et al 2016 [46]	Markov model	Cost-utility analysis	Switzerland third-party payer	Direct health costs	2016 CHF	Univariate and probabilistic

Abbreviations: Swiss francs (CHF); Randomized controlled trial (RCT); United States (US); United Kingdom (UK)

Supplementary Table S2. Quality appraisal of individual included studies according to GRADE scale

Study Reference, year	Quality appraisal (GRADE scale) (n=59 studies)	
	**Scoring**	**Appraisal**
Barbier MC et al., 2021 [9]	High<br>⊕⊕⊕⊕	The true effect lies close to that of the estimate of the effect.
Chang WC et al. 2021 [67]	Moderate<br>⊕⊕⊕◯	Rarity of disease. Such low disease prevalence may result in heterogeneity of disease diagnosis and treatment effect.
Cheng S et al 2021 [33]	High<br>⊕⊕⊕⊕	The true effect lies close to that of the estimate of the effect.
Liu X et al 2021 [62]	Moderate<br>⊕⊕⊕◯	Limitation related to generalizability.
Liu G et al 2021[34]	Moderate<br>⊕⊕⊕◯	The effect estimate as the true effect is likely to be close to the estimate of the effect, but there is a possibility that it is quite different.
Hu H et al 2021 [12]	Moderate<br>⊕⊕⊕◯	The effect estimate as the true effect is likely to be close to the estimate of the effect, but there is a possibility that it is quite different.
Shi Y et al 2021 [68]	Moderate<br>⊕⊕⊕◯	The effect estimate as the true effect is likely to be close to the estimate of the effect, but there is a possibility that it is quite different.
Cai Y et al 2021 [35]	High<br>⊕⊕⊕⊕	The true effect lies close to that of the estimate of the effect
Qin S et al 2021 [47]	High<br>⊕⊕⊕⊕	The true effect lies close to that of the estimate of the effect
Peng Y et al 2021 [48]	Moderate<br>⊕⊕⊕◯	Limitation related to generalizability.
Qiao L et al 2021 [13]	Moderate<br>⊕⊕⊕◯	Bias in the economic results.<br>The effect estimate as the true effect is likely to be close to the estimate of the effect.
Yang SC et al 2021 [36]	Moderate/High<br>⊕⊕⊕◯	The effect estimate as the true effect is likely to be close to the estimate of the effect.
Wurcel V et al 2021 [56]	Moderate<br>⊕⊕⊕◯	The effect estimate as the true effect is likely to be close to the estimate of the effect, but there is a possibility that it is quite different.
Insinga RP et al 2021 [11]	Moderate/High<br>⊕⊕⊕◯	The effect estimate as the true effect is likely to be close to the estimate of the effect.
Peng Y et al 2021 [37]	Moderate/Low<br>⊕⊕◯ ◯	The effect estimate as the true effect is likely to be close to the estimate of the effect, but there is a possibility that it is substantially different.
Liu Q et al 2021 [14]	Moderate/High<br>⊕⊕⊕◯	The effect estimate as the true effect is likely to be close to the estimate of the effect.
Panje CM et al 2020 [15]	Moderate/High<br>⊕⊕⊕◯	The effect estimate as the true effect is likely to be close to the estimate of the effect.
Wan N et al 2020 [38]	Moderate/High<br>⊕⊕⊕◯	The effect estimate as the true effect is likely to be close to the estimate of the effect.

Weng X et al 2020 [63]	Moderate/Low<br>⊕⊕◯ ◯	Limitation related to generalizability.<br>The effect estimate as the true effect is likely to be close to the estimate of the effect, but there is a possibility that it is substantially different.
**Study Reference, year**	**Scoring**	**Appraisal**
Weng X et al 2020 [16]	Moderate<br>⊕⊕⊕◯	The effect estimate as the true effect is likely to be close to the estimate of the effect, but there is a possibility that it is quite different.
Wu B et al 2020 [64]	Moderate<br>⊕⊕⊕◯	The effect estimate as the true effect is likely to be close to the estimate of the effect, but there is a possibility that it is quite different.
Zhou K et al 2020 [57]	Moderate/High<br>⊕⊕⊕◯	The effect estimate as the true effect is likely to be close to the estimate of the effect
Criss SD et al 2020 [39]	Moderate/High<br>⊕⊕⊕◯	The effect estimate as the true effect is likely to be close to the estimate of the effect
Lauren B et al 2020 [69]	Moderate/High<br>⊕⊕⊕◯	The effect estimate as the true effect is likely to be close to the estimate of the effect
Liu Q et al 2020 [40]	Moderate/High<br>⊕⊕⊕◯	The effect estimate as the true effect is likely to be close to the estimate of the effect
Parmar A et al 2020 [54]	Moderate/High<br>⊕⊕⊕◯	The effect estimate as the true effect is likely to be close to the estimate of the effect
Rothwell B et al 2020 [17]	Moderate/High<br>⊕⊕⊕◯	The effect estimate as the true effect is likely to be close to the estimate of the effect
Li J et al 2020 [18]	Moderate/High<br>⊕⊕⊕◯	The effect estimate as the true effect is likely to be close to the estimate of the effect
Aziz MIA et al 2020 [41]	Moderate<br>⊕⊕⊕◯	The effect estimate as the true effect is likely to be close to the estimate of the effect, but there is a possibility that it is quite different.
Hale O et al 2020 [49]	Moderate<br>⊕⊕⊕◯	The effect estimate as the true effect is likely to be close to the estimate of the effect, but there is a possibility that it is quite different.
Wu B et al 2020 [19]	Moderate/High<br>⊕⊕⊕◯	The effect estimate as the true effect is likely to be close to the estimate of the effect.
Patterson K et al 2019 [50]	Moderate/High<br>⊕⊕⊕◯	The effect estimate as the true effect is likely to be close to the estimate of the effect.
Reinhorn D et al 2019 [53]	Moderate/High<br>⊕⊕⊕◯	The effect estimate as the true effect is likely to be close to the estimate of the effect.
Longjiang She et al 2019 [20]	Moderate/High<br>⊕⊕⊕◯	The effect estimate as the true effect is likely to be close to the estimate of the effect.
Wan X et al 2019 [42]	Moderate/High<br>⊕⊕⊕◯	The effect estimate as the true effect is likely to be close to the estimate of the effect.
Zhou K et al 2019 [43]	Moderate/High<br>⊕⊕⊕◯	The effect estimate as the true effect is likely to be close to the estimate of the effect.
Bhadhuri A et al 2019 [21]	Moderate/High<br>⊕⊕⊕◯	The effect estimate as the true effect is likely to be close to the estimate of the effect.

Chouaid C et al 2019 [22]	Moderate/High ⊕⊕⊕◯	The effect estimate as the true effect is likely to be close to the estimate of the effect.
Criss SD et al 2019 [51]	Moderate/High ⊕⊕⊕◯	The effect estimate as the true effect is likely to be close to the estimate of the effect.
Huang M et al 2019 [23]	Moderate/High ⊕⊕⊕◯	The effect estimate as the true effect is likely to be close to the estimate of the effect.
**Study Reference, year**	**Scoring**	**Appraisal**
Insinga RP et al 2019 [31]	Moderate/High ⊕⊕⊕◯	The effect estimate as the true effect is likely to be close to the estimate of the effect.
Liu M et al 2019 [58]	Moderate ⊕⊕⊕◯	Limitation related to generalizability.
Loong HH et al 2019 [30]	Moderate/High ⊕⊕⊕◯	The effect estimate as the true effect is likely to be close to the estimate of the effect.
Liao W et al 2019 [44]	Moderate/Low ⊕⊕◯ ◯	The effect estimate as the true effect is likely to be close to the estimate of the effect, but there is a possibility that it is substantially different.
Meng Y et al 2018 [65]	Moderate/High ⊕⊕⊕◯	The effect estimate as the true effect is likely to be close to the estimate of the effect.
Sarfaty M et al 2018 [52]	Moderate/High ⊕⊕⊕◯	The effect estimate as the true effect is likely to be close to the estimate of the effect.
Sarfaty M et al 2018 [55]	Moderate/High ⊕⊕⊕◯	The effect estimate as the true effect is likely to be close to the estimate of the effect.
Tarhini A et al 2018 [66]	Moderate/High ⊕⊕⊕◯	The effect estimate as the true effect is likely to be close to the estimate of the effect.
Zargar M et al 2018 [59]	Moderate/High ⊕⊕⊕◯	The effect estimate as the true effect is likely to be close to the estimate of the effect.
Aguiar P et al 2018 [45]	Moderate ⊕⊕⊕◯	The effect estimate as the true effect is likely to be close to the estimate of the effect, but there is a possibility that it is quite different.
Hirschmann A et al 2018 [60]	Moderate/High ⊕⊕⊕◯	The effect estimate as the true effect is likely to be close to the estimate of the effect.
Hu x et al 2018 [32]	Moderate/High ⊕⊕⊕◯	The effect estimate as the true effect is likely to be close to the estimate of the effect.
Insinga RP et al 2018 [25]	Moderate/High ⊕⊕⊕◯	The effect estimate as the true effect is likely to be close to the estimate of the effect.
Georgieva M et al 2018 [26]	Moderate/High ⊕⊕⊕◯	The effect estimate as the true effect is likely to be close to the estimate of the effect.
Ward MC et al 2017 [61]	Moderate/High ⊕⊕⊕◯	The effect estimate as the true effect is likely to be close to the estimate of the effect.
Aguiar PN Jr et al 2017 [27]	Moderate ⊕⊕⊕◯	The effect estimate as the true effect is likely to be close to the estimate of the effect, but there is a possibility that it is quite different.
Huang M et al 2017 [29]	Moderate/High ⊕⊕⊕◯	The effect estimate as the true effect is likely to be close to the estimate of the effect.

Matter-Walstra K et al 2016 [46]	Moderate/High<br>⊕⊕⊕◯	The effect estimate as the true effect is likely to be close to the estimate of the effect.
